# ‘Birthing a Better Future’: A mixed‐methods evaluation of an exhibition on the early years of life

**DOI:** 10.1111/hex.13259

**Published:** 2021-05-01

**Authors:** Maya Lakhanpaul, Emma C. Alexander, Meghan A. Cupp, Jessica Taripre Owugha, Alex Florschutz, Andy Beckingham, Virad Kisan, Monica Lakhanpaul, Logan Manikam

**Affiliations:** ^1^ University of Manchester Manchester UK; ^2^ London North West University Healthcare NHS Trust London UK; ^3^ King's College London London UK; ^4^ UCL GOS Institute of Child Health London UK; ^5^ Aceso Global Health Consultants Limited London UK; ^6^ Zero2 Expo East Sussex UK; ^7^ Fernandez Foundation Hyderabad India; ^8^ UCL Medical School London UK; ^9^ Whittington Health NHS Trust London UK; ^10^ UCL Institute of Epidemiology and Health Care London UK

**Keywords:** health education, paediatric and infant health, public health, visual arts

## Abstract

**Background:**

Our study aimed to evaluate to what extent Zero2 Expo's ‘Birthing a Better Future’, a co‐created multimedia exhibition, was effective in raising awareness on the importance of the first 1001 days of life and explore what refinements would help to optimize the impact of future exhibitions.

**Methods:**

We conducted a mixed‐methods evaluation of the exhibition delivered in the John Radcliffe Hospital, Oxford. Through convenience sampling, 14 participants were selected to participate in 12 structured interviews and 19 participants completed a questionnaire. Interviews were thematically analysed alongside quantitative analysis of questionnaire responses through Likert scales.

**Results:**

The majority (78.6%, n = 11/14) of participants who completed the questionnaire either agreed or strongly agreed that the exhibition raised their awareness about the first 1001 days of life. This was supported by the analysis of interviews. The use of art was found to provoke an emotional engagement from participants. Participants felt that the length of the written pieces and location of the exhibition were important factors for designers to consider in future exhibitions.

**Conclusion:**

This study demonstrated that multimedia exhibitions, combining science with art, may be an effective way to raise awareness of public health messages. Engaging with key stakeholders will be an essential step in order to improve future public health exhibitions.

**Public Contribution:**

When designing the study, the public reviewed the study tools, which were refined based on their feedback. At every phase of the study, members of the public who are artists co‐created the exhibition content.

## 1 INTRODUCTION

Early years of life are pivotal for child development. Only a quarter of the upper brain (cerebral cortex) is developed in humans by the time of birth. In addition, the prefrontal and anterior cingulate, temporal (visual) cortex and hippocampus are all considered to be primitive at birth. The first years of life are therefore critical since this is the period where the brain is evolving and expanding at the greatest speed.^1^ This is due to both biological mechanisms, such as gene expression, and environmental influences: nutrition, environmental stimuli and sufficient nurturing from adults, which helps to stimulate emotional and social connections.^2^ Although the human brain continually develops throughout the lifetime, much of its structure and capacity are established before the age of two. Inability to optimize early brain development can have long‐term consequences on educational attainment, job potential and adult mental health.^3^

Public health interventions targeting early childhood health and development are therefore thought to hold great potential for return on investment.^4^ This has been recognized politically, with the United Kingdom's (UK) first cross‐party children's manifesto ‘1001 Critical Days—from Conception to Age Two’ (‘the manifesto’), launched in 2013. The manifesto emphasizes a holistic approach with the aim to support parents and health‐care professionals in ensuring that babies are optimally cared for. In regard to parents of young children, it suggests educating them about the criticality of the first 1001 days, alongside focusing on the accessibility of services for parents, such as antenatal classes and mental health services. In addition, it promotes the collaboration of professionals across multiple sectors so that patients are seen appropriately, and it seeks to ensure that those working in the profession are adequately trained.^5^ The manifesto therefore sets out a vision for service provision in early years across the UK, highlighting the moral, scientific and economic importance of this crucial period.^5^ Multiple organizations and public figures have since pledged their support of the manifesto, such as the NHS Lambeth & Leeds Clinical Commissioning Groups.

Separately, the role of art in science and technology fields is being increasingly promoted^6^, ^7^, with conceptual shifts from a focus on STEM to STEAM (science, technology, engineering, arts and mathematics).^8^ However, further efforts are still required to improve integration of the arts and health‐care sectors. Art therapy in health care has been explored as an avenue to assist in the treatment of patients^9^ and a healing intervention for children in a variety of contexts, especially relating to psychiatric disorders and cancer.^10^ However, there is limited evidence with regard to research on the effect that art can have on complementing public health methods to deliver messages to different populations.^11^

The Zero2 Expo initiative began as a pilot art and science multimedia exhibition in the Houses of Parliament in 2016, inspired by the manifesto. Subsequently, the ‘Birthing a Better Future’ interdisciplinary exhibition (‘the exhibition’) was co‐created with the aim of showcasing an educational, multimedia art and science exhibition, raising awareness of the first 1001 days of life.^12^ It was particularly designed to hold relevance for the general public, as well as stakeholders and service providers in child health. This exhibition was first showcased at the John Radcliffe Hospital in 2017 to increase public awareness about the key issues and importance of these first 1001 days of life. This paper reports on an evaluation of this exhibition at the John Radcliffe Hospital.

We therefore conducted this study to evaluate the effectiveness of the exhibition at raising awareness of the importance of the first 1001 days of life. The specific evaluation aims were to monitor the engagement with the exhibition, assess the impact of the exhibition on public awareness and knowledge of the first 1001 days of life, and evaluate the effectiveness of a multimedia art and science approach on public engagement.

Secondarily, we collected feedback regarding what refinements the target audience would suggest to improve the content, format and delivery of the exhibition to enhance engagement and inform future exhibitions.

## 2 METHODS

### 2.1 Context

**FIGURE 1:**
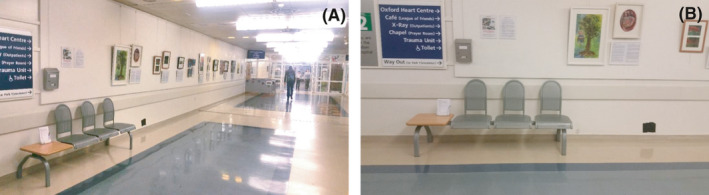
A; Birthing a Better Future Exhibition at the Corridor Gallery, John Radcliffe Hospital, Oxford. The gallery is situated on the ground floor between the main entrance and café. Art and written pieces displayed on the left corridor wall, as heading into the hospital. B; Location of the questionnaire forms. Location of the questionnaire forms and pen relative to the exhibition with pen, on the table in the seating area of the gallery. Completed forms were deposited in the secured postbox on the wall. (Photo credit: Jessica T. Owugha)

The exhibition's opening showcase was held between 17:00 and 19:00 hours on 11 November 2017, only being available to invited guests. Following the showcase, the exhibition was then hosted in the corridor gallery of the John Radcliffe Hospital, Oxford, from 12 November 2017 to 6 January 2018 (Figure 1A), with a plan to roll out the exhibition across the UK and internationally into 2020. This location was chosen following an invitation to host at the John Radcliffe Hospital, Oxford.

The exhibition included visual and written art pieces by nine artists (topics listed in Table 1), inspired by conversations with scientists about the critical period of child development and based on the underlying themes in the 1001 days' report. The artists chose the theme of their artwork based on their own personal experiences and emotional connection, such as postnatal depression, and carried out their own research on that subject matter. To fulfil the project aim for a multimedia exhibition, the art pieces were complemented by written information provided by academics with expertise on the subject matter portrayed in the artwork. Studies have indicated that an intervention that is multifaceted, although costly to produce, may be more impactful than an intervention, which utilizes one delivery mode only, such as written articles.^13^ This is because it is believed that art can help to provoke emotion about the topic being explored above that which may be achieved through written information alone. In the light of this, written pieces (see Table 2), informed by evidence, were provided to add credibility and provide an explanation of the messages presented by the art pieces.

**TABLE 1:** Art pieces presented at exhibition

Title of art piece	Artist
The fabric of life	
The fabric of young life	Barbara Shaw
Suspundu	
Beginnings	Charlie Henry
Baby brain, variation #1	Georgina Griffiths
The second trimester	
Helena	Joanne Makin
Domestic Bliss	Gill White
Johnny	Nia Walling
Touching movements	
Underwater dreams	Helen Edwards
Held with light (series 1‐3)	Melissa Sheffield
Life before birth	
In the beginning	Alex Florschutz

**TABLE 2:** Written pieces presented at exhibition

Title of written pieces	Artists
Homelessness in the perinatal period	Dr Alice Haynes
Impact of creative interventions in postnatal depression	Dr Rosie Perkins
Domestic bliss/Domestic abuse ‐ protective factors from	Jonathan Rawlings
Neurobiology, brain connections and relatedness	Dr Karen Bateson
Infants' motor, social and cognitive learning from age 0 to 2	Dr L. Rat‐Fischer
The importance of the time on the womb	Professor Vivette Glover

### 2.2 Participant sampling

All participants for the interviews were selected from those engaging with the exhibition through convenience sampling at specific times of the day when the research fellow (JO) was present for recruitment. In this study, engagement was defined as a pause or change in walking trajectory where attention is diverted towards one or more exhibition piece(s). Of those individuals who were observed engaging with the exhibition, some had been invited by organizers to the showcase, and were therefore attending with the purpose of specifically visiting the exhibition, whilst others were just passing through the hospital corridor where the exhibition had been set up. The researcher, however, was not aware of whether the participant had been invited to the exhibition or was just a passer‐by, prior to conducting the interview. All ages were eligible (with parental consent required for children). There were no exclusion criteria apart from not being able to speak, or read, in English. In addition, quantitative data were collected using freely available questionnaires, which were provided on a table in the seating area of the gallery during the entire exhibition period.

### 2.3 Data collection

We conducted a mixed‐methods evaluation of the exhibition. Questionnaires were used to collect quantitative and qualitative data for evaluating the exhibition, whilst structured interviews were used to gather only qualitative data. Similar questions were included in the questionnaires, and interview prompts to help provide a rounded perspective and triangulate data. These interview questions were piloted with participants during the opening showcase night, alongside the questionnaire that was piloted with five participants. The questions that were removed from the pilot questionnaires and interviews were not included in the final data analysis. Changes to the guide and questionnaire following the piloting feedback are presented in the Appendix 1.

The use of questionnaires allowed for collected data to be systematically compared^14^ since a majority of the questions relied on participants indicating their level of agreement or disagreement with a statement about the exhibition using a Likert scale. These questionnaires can be seen in the Appendix 1 (Likert statements: ‘The exhibition was thought provoking’, ‘The exhibition has increased my a) awareness b) knowledge of the first 1001 days of a child's life’). Two free‐text questions, where participants wrote unstructured responses to prompts, were also included. Participants were asked to self‐identify their backgrounds from five categories: ‘general public’; ‘parents or carers’; ‘health‐care professional’; ‘maternal and child health policymaker’; and ‘maternal and child health sector organization members’. Participants then deposited their completed questionnaires into a secured postbox mounted on the wall beside the exhibition's introductory piece (Figure 1B).

Additional data were collected by an onsite researcher (JO) between the hours of 09:30 and 17:30 on a randomly selected subset of 6 days across the exhibition period, covering 10% of the total exhibition duration, excluding public holidays. On days when JO was present, engaged individuals walking through the gallery were first offered an interview, and if they declined, they were invited to complete quantitative questionnaire instead. Participants who completed an interview were not also offered a questionnaire after the interview. Structured interviews were beneficial for this study since they enabled the researcher to develop an in‐depth understanding of the participants' thoughts, feelings and experiences of the exhibition through the use of more open‐ended questions.^14^ The interviews were conducted by JO, a researcher trained in Patient and Public Involvement (PPI) facilitation and discussion. JO conducted interviews with fourteen individuals. Of these, two interviews contained two participants, therefore leading to a total of 12 interviews being conducted. We are aware of varying guidance on sufficient sample sizes for data saturation, and followed guidance advising that a sample size of 12 or more would be sufficient.^15^ This study's sample size of 14 interview participants, supplemented by respondents completing questionnaires, was therefore thought to be sufficient to attain data saturation for the scale of this particular study. All interviews took place in the Corridor Gallery. Audio recordings of the interviews were captured using an Olympus DM‐650 16‐Bit Stereo Recorder and transcribed verbatim.

### 2.4 Data analysis

Questionnaire responses were collated and assessed using the R software version 1.1.423 with the ‘Likert’ package. The free‐text responses from the questionnaire were qualitatively assessed by JO into themes by using interpretive phenomenological analysis. The interview transcripts, independent from the free‐text responses, were also analysed thematically and coded by an independent researcher, ML. ML and JO used the interpretative phenomenological analysis to establish common themes from the interview transcripts utilizing the process of constant comparison. The interpretative phenomenological analysis was used because the aim of the study was to deductively determine themes that arose from the participants' words themselves and not to evaluate a pre‐existing theory.^16^ Furthermore, thematic analysis is an effective methodological tool to develop a comprehensive understanding of the data and distinguish specific themes derived inductively from the data itself instead of from the researcher's own ideas, and was hence most appropriate for this study.^17^

### 2.5 Ethics

The HRA decision tool^18^ indicated that approval from the NHS REC, for sites in England, was not necessary for this particular study. In addition, we sought advice from the UCL ethics team who concluded that, since the exhibition was a public engagement activity and deemed low risk, formal approval was not required. Permission from the Oxford Hospitals Charity for the Oxford University Hospitals NHS Foundation Trust was, however, obtained to hold the exhibition at the John Radcliffe Hospital and evaluate it accordingly. In addition, in line with the best practice, informed consent was obtained for the structured interviews by explaining the aims and procedures of the study to the participant as per the information sheet. Written informed consent was then obtained and verbal consent confirmed on commencement of the interview. Since one of the participants was younger than 16 years, consent was also obtained from an accompanying adult. Following transcription of the interviews, the participants' names were then anonymized to maintain participant confidentiality. Digital sound files were also issued a participant code and stored in an encrypted file on a secure computer in order to protect the data.

## 3 RESULTS

**FIGURE 2:**
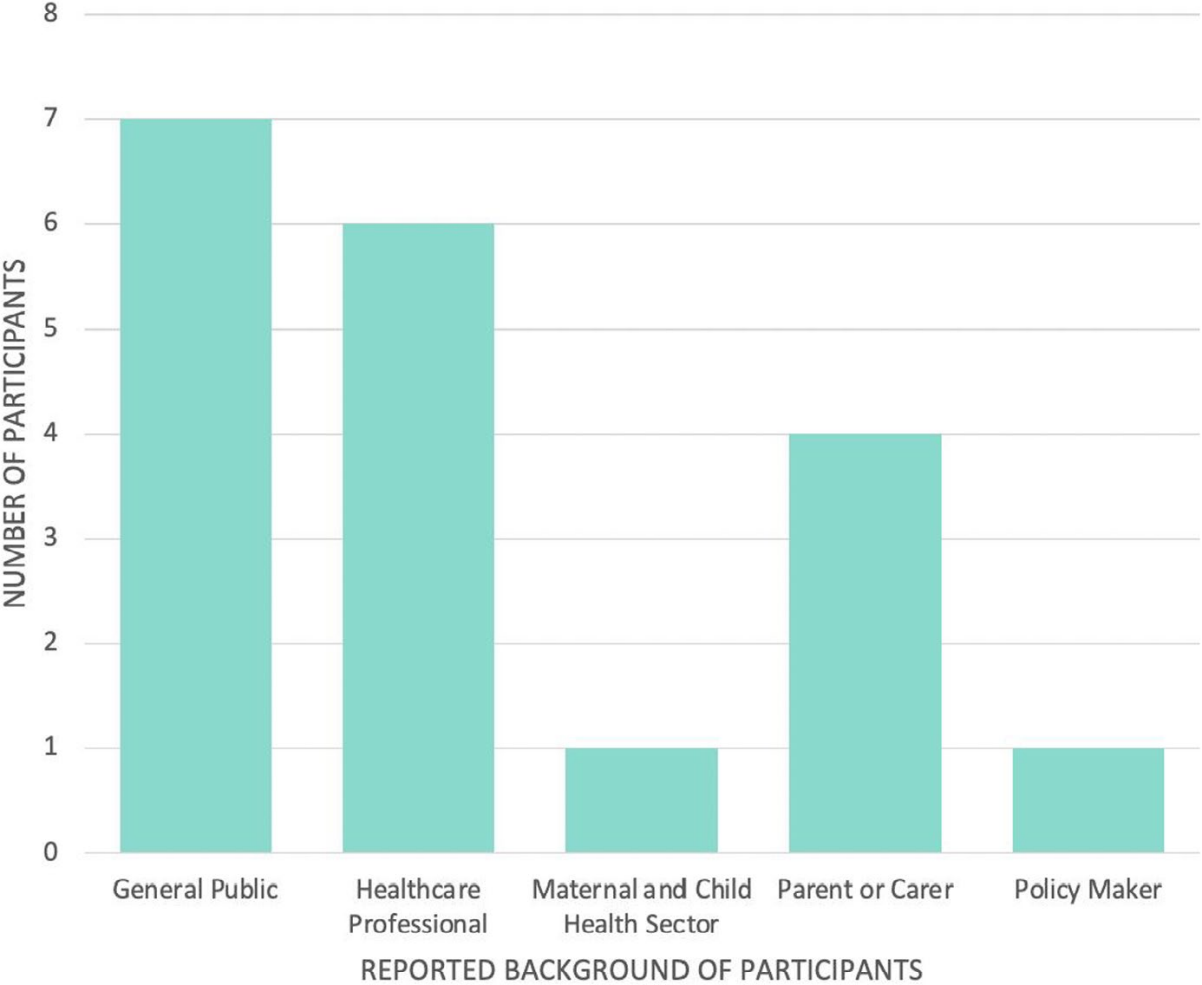
Participant demographics for the questionnaires

**FIGURE 3:**
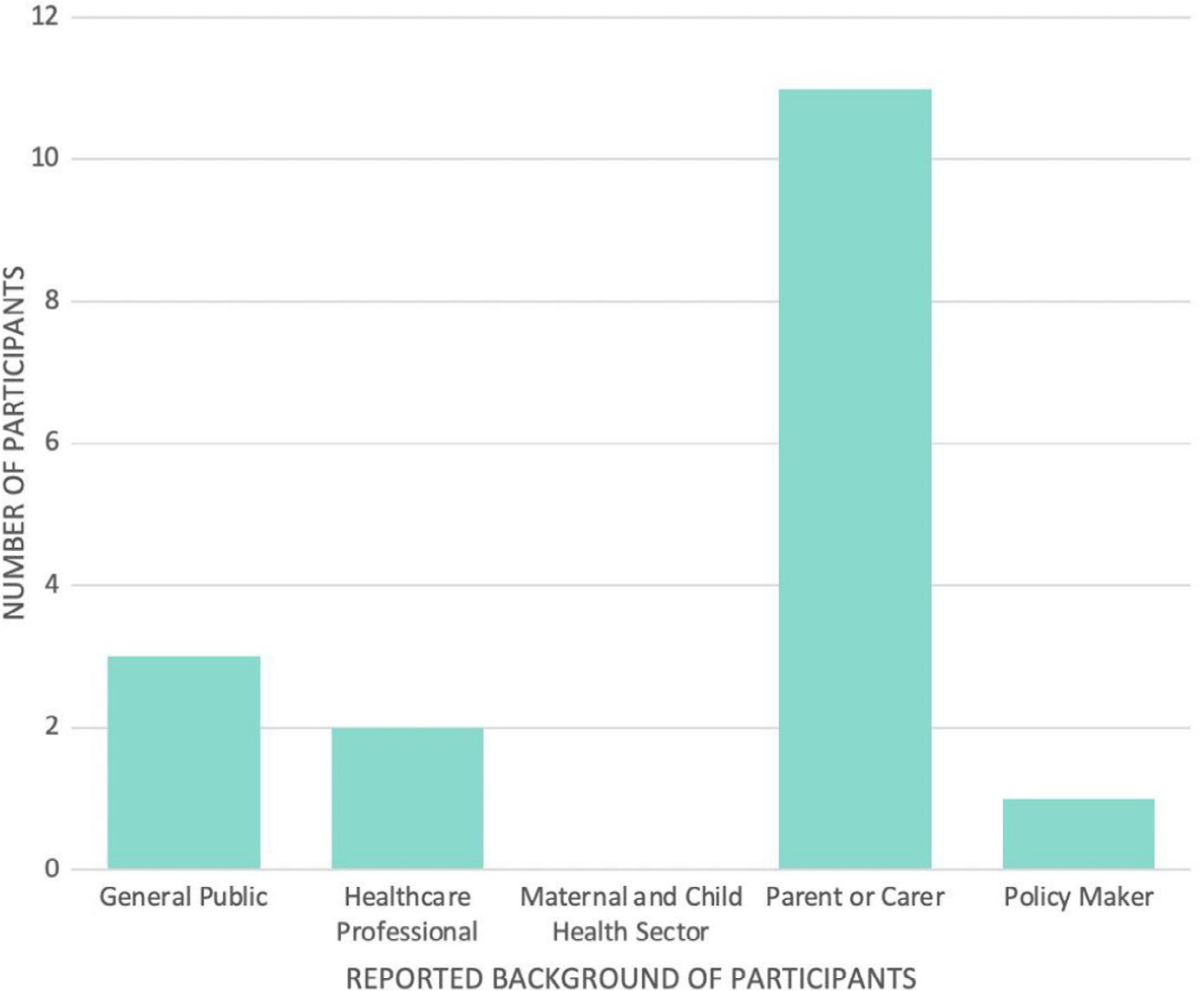
Participant demographics for the interviews

Fourteen questionnaires were collected following the five that were completed during the pilot test on the opening showcase night; the majority of respondents identified as either members of the general public (36.8%, n = 7/19) or health‐care professionals (31.6%, n = 6/19), rather than experts in child health (Figure 2). In total, there were 14 interview participants, with the majority of participants self‐identifying as parents (78.6%, n = 1114); however, unlike the questionnaire where participants only identified with one category, some interview participants dually identified (Figure 3).

Analysis of the structured interviews and questionnaire data revealed two central themes relevant to the exhibition, which are discussed below. These themes included the following: (a) impact of the exhibition and (b) how to increase the impact of public health–related exhibitions.

### 3.1 Impact of the exhibition

The impact of the exhibition was highlighted both through the questionnaire responses and through structured interviews. The majority of participants felt that viewing the exhibition increased awareness about the importance of the first 1001 days of a baby's life and also triggered an emotional response. These two subthemes are discussed below.

#### 3.1.1 Increased awareness

Participants reported that the exhibition contributed to new knowledge and heightened awareness of the importance of the early years of life. For example, this was emphasized through one participant's recognition of the exhibition's message and its novelty:

I had no idea about the importance of the first 1000 days for a child's brain development. [This was] new [information] for me. **Parent, healthcare professional**

Participants further discussed specific aspects of the exhibition's key message and acknowledged the impact of the first 1001 days of a child's life, drawing their own conclusions from the messages presented by the exhibition. One participant discussed an increase in their own awareness and the importance of childhood exposures on later life:

What I became…very well aware [of], is how the beginning of the life of a human being which is very malleable. And, therefore, there are a lot of factors which impinge on it. These are exceptionally important, and this could shape the life and the future. **Parent**

Increased knowledge of the highly impressionable period that children experience in the early years of life was also reported by participants. For example, one participant who self‐identified as a parent but was also an artist that contributed to the exhibition discussed the exhibition in the context of abuse and acknowledged that it is a vital issue for child health, which may be under‐recognized:

…the piece of work about the domestic violence. That has really highlighted how prolific it is. **Parent**

**FIGURE 4:**
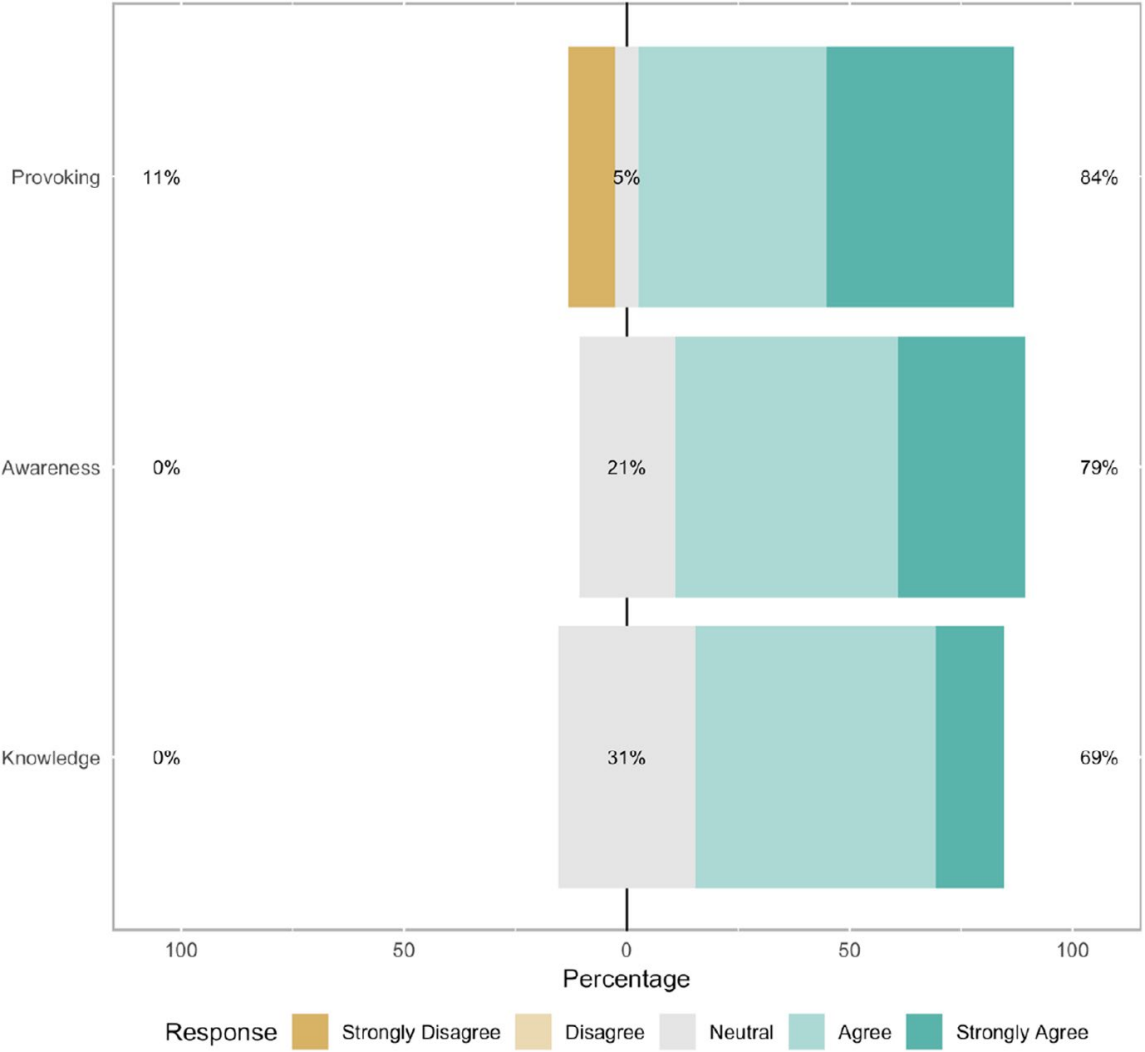
Likert‐scale results

The notion that the exhibition increased awareness of one's knowledge about the criticality of the first 1001 days of one's life was supported by the questionnaire data. The questionnaire data indicated a consistently positive response to the exhibition (Figure 4) with 50.0% (n = 7/14) of respondents agreeing that the exhibition raised their awareness of the topic and 28.6% (n = 4/14) strongly agreeing with this statement. In addition to this, 64.3% (n = 9/14) of participants felt that the exhibition affected their knowledge.

#### 3.1.2 Emotional engagement

The exhibition also reinforced participants' existing ideas around the importance of showing love and affection, particularly towards children. For example, one participant describes how the exhibition triggered a desire to spend more time with her daughter:

There were some photos and I found those particularly moving. The pictures of mother and child close together. That really hit a nerve for me… It just makes me want to go home and cuddle my daughter… have more skin‐to‐skin […] and give into those urges… **Parent**

In addition, the exhibition revealed discussions about more complex issues surrounding childbirth and the first 1001 days, especially with regard to the negative and challenging aspects. The excerpt below, from an interview participant whose art work was also displayed at the exhibition, summarizes this concept:

The message […] those feelings are all part of birthing and the anxieties around it. I was just talking to the mother of a woman who said that she had had some difficulties. And I remember that I had that as well but being very surprised whereas you feel with your first child that it would be the most beautiful and wonderful thing. But it's not necessarily ‐ there's all that ambivalence that goes with it. **Parent**

Both quotes illustrate that the exhibition not only conveyed the intended message but also allowed participants to engage emotionally with the importance of the first 1001 days.

### 3.2 How to increase the impact of public health–related exhibitions

Data from the study highlighted that practical factors are important in order to optimize the impact of public health–related exhibitions, in particular the location of the exhibition and the use of a multimedia approach. These two subthemes are discussed below.

#### 3.2.1 The location of the exhibition

The exhibition location, a hospital corridor, was considered by some participants to be an appropriate and effective place for the exhibition by some participants:

I think it's really good to have [some] a subject matter that's relevant in the corridor in a hospital… I think a relevant thing is that it's an audience who would not normally go into a gallery or a space showing art. **Parent**

However, it was acknowledged by a majority of participants that due to hospitals being a busy and stressful environment, the exhibition may be overlooked by people passing. Participants therefore suggested alternative settings where people can take in the content of the exhibition in a more relaxed setting:

Public places of course, where people can stop and read… large shopping places where people can rest or a cafeteria where people can chill out, relax and spend a little bit of time. **Parent, healthcare provider**

People come here, and I came here for a specific reason. This is a corridor. This is a hospital. As you can see I went through Accident and Emergency today and I haven't slept for 3 days and 3 nights. **Parent**

Maybe perhaps in a general area… when visitors have got time to pursue, rather than it being shown in the main thoroughfare 'cos everyone is busy trying to get to their appointment. **General public**

One participant, whose artwork was displayed in the exhibition, even suggested specific locations that they believed may be effective to reach audiences that may also be more difficult to access:

I think that sometimes with infant care the focus is really on the mum… I think that it's really important that dads are included because dads are important. It could reach children's centres and things like that where there's maybe a group [of people] at that point. **Parent**

You know taking it directly into a maternity ward.. I wanted to also take my piece into say like a men's club.. where men actually might stop and look and think. **Parent**

The interviews therefore suggest that the location of the exhibition should be reconsidered or tailored for the audience it is trying to reach.

#### 3.2.2 The use of a multimedia approach

There were notable differences in opinion as to the use of a multimedia exhibition in this context. Some participants expressed concerns about the principle of using artwork to convey a message.

Some of the work was engaging. Nonetheless, artworks can be limiting in terms of understanding what they portray. **General public**

Even so, the use of art to portray a public health message was considered to be positive by other interview participants. Participants enjoyed seeing artwork with meaning behind it, as opposed to usual artwork they perceived as ‘meaningless’:

… obviously the image is more than a 1000 words… I tend to be captured more by images. **Parent**

… the art is good. Art is brilliant. Art is excellent.**​** **Parent**

… very good media to convey the message. **Parent**

The artwork was also praised for being accessible to different audiences:

… you don't necessarily need to be able to read. If say English wasn't your first language, or you couldn't even understand the English, you could look at it perhaps make something of it. **General public**

…I think that raising awareness…particularly through art and the idea that art like this might go out there in the world…may be that's a good way to reach different people in a less direct or lecturing way. **Parent**

Participants, however, felt that the artwork was more ‘powerful’ (feedback from parent) than the piece of text accompanying the work, since some believed the text to be too long:

To me, text is a bit boring […]. Especially in a hospital when you're flying through. You 'aint got time to sit there and read, but when you see the visual pieces you're like, wow(!), but you haven't got time to read. **Parent**

Less text. […] it is an awful lot to read […] I felt as though it was too long winded and [I] just wanted to know what was going through the artists' eyes really. **General public**

It's incredibly lengthy, they probably put you off. **General Public**

Some participants also believed that some of the scientific text was too detailed and complex:

… because there is a lot of jargon, unless you've studied psychology or sociology you won't really have an understanding… sometimes it goes into a lot more detail, it doesn't always perceive what you see in the picture I think. **General public**

Moreover, the combination of artwork alongside written pieces was considered by participants to be an effective method to portray information about the first 1001 days of a baby's life.

…I think it's great. It's very informative and […] you did a good job with a combination of both [visual and written pieces]. **Parent, healthcare provider**

To put text and artwork together links them so difficult to separate them. **General public**

The same respondent suggested that the exhibition could be enhanced through the incorporation of three‐dimensional pieces:

The installation by [Artist] would have been good to see for real and not as photo. **General public**

## 4 DISCUSSION

Overall, the co‐created exhibition received positive responses with research participants reporting favourably on the work exhibited and the underlying concepts behind the artwork. The interviews demonstrated how use of an exhibition may be effective in raising awareness of a public health issue ‐ in this case, the first 1001 days of life. Although the participants reported that they enjoyed the multimedia aspects of the exhibition with the combination of both art and text, this study further highlighted that considerations should be given to the location of the exhibition alongside the length of the written pieces. Additionally, this study provides another example of the utility of art as a method to engage and communicate with the public and provides learning points and insights or future public health professionals planning similar campaigns.

There are a number of points of discussion from this study. Although it may be hard to convey raw data and statistics in an artistic form, our study found that art and written communication in this setting may be a valuable way to not only communicate with an audience but also stimulate their emotions. Studies that have assessed the effectiveness of anti‐smoking campaigns have shown that media that triggers emotion may be more effective since it generates discussion and attention.^19^ In addition to this, some studies have shown that the type of emotion elicited may also play a vital role. Montazeri and McEwen^20^ found that mass‐media campaigns for anti‐smoking that stimulated fear were more effective than those that triggered positive feelings. Participants in the present study reported emotive responses of both fear and love, elicited by the artwork related to child abuse and the art depicting a mother and daughter, respectively. The long‐term impact of the provocation of these two emotions in relation to the effectiveness of the exhibition is, however, beyond the scope of this evaluation.

Our evaluation also highlighted the delicate balance between art and written text in mixed‐media exhibitions, where it is essential that the written text is as engaging as the artwork. Some participants reported that the amount of text presented in the current iteration of the exhibition, around two sides of A4 size paper, was too long and at times too complex. Studies have highlighted that simpler text that is modified to be understood by a particular target audience, such as a lay audience, is more likely to be understood and therefore to be more effective.^21^ This was demonstrated in a study that analysed parents' opinions on a fever guidebook.^22^ The study found that parents preferred text that was easily digestible and under one page. More research is, therefore, needed to determine the optimum layout for this type of exhibition. A participatory approach, in which the exhibition is co‐developed with the target audience, should be considered for future exhibitions to tailor language and content.

There are diverse views on whether placing an exhibition in an area of high thoroughfare, such as the hospital corridor, is the most optimal environment. There was mixed feedback, with some participants responding favourably to the setting and others being less positive. The location of the exhibition, approximately 200 m away from the hospital entrance, was seen as accessible since it was in a public domain and not far to walk. The use of a health‐care setting was also thought by some to be beneficial as it enabled health‐care professionals to engage in the exhibition and reflect on the issues raised in the context of their professional practice. However, several disadvantages to using the hospital corridor were reported. As the majority of people in a hospital are either unwell or visiting someone who was sick, it is possible that they may not be in the most receptive frame of mind for receiving a public health message. This reflection is supported by a systematic review on ‘information resources to aid parental decision‐making on when to seek medical care for their acutely sick child’.^23^ The study found that interventions carried out in emergency departments were less successful than those carried out in a setting that is non‐stressful, such as one's home. This may be because research has shown stress can have a significant impact on learning.^24^ Other locations where individuals may have the time and capacity to absorb the information portrayed in the exhibition should be considered in future.

The nature of the location also restricted the type of artwork that could be presented. For example, an original installation and an audio piece were adapted to fit the constraints of exhibiting in a hospital. Research has shown that when given both written and verbal materials, a person is more likely to remember it for a longer period of time, as opposed to written data alone.^23^ For future exhibitions, it may therefore be beneficial to take this into account and contemplate whether a location that can accommodate audio pieces or installations, as suggested by one participant, may be more effective. Further to this, if the piece was in an audio format, perhaps the length of the written pieces may have presented less of a barrier. The value of physical artwork compared with digital presentation warrants further investigation.

Following the findings of this evaluation, Zero2 Expo has developed a revised pop‐up exhibition model targeted in particular towards parents and the general public. This exhibition has begun to be displayed around the UK, and further evaluation will follow shortly.

### 4.1 Limitations of the study

It is important to note that a major limitation of our study was the sampling method. Although agreement by participants who were approached for interview was high, there were only a small number of questionnaires and interviews elicited relative to the overall number of people passing through the exhibition. This may mean that key themes that may be relevant to the audience at large were not captured from the interviews and questionnaires that we conducted. We note that, using the methodology from Guest et al^25^ for evaluating data saturation, we achieved a new information threshold of 10%, which is greater than the target of <5%. Nevertheless, data saturation is affected by a number of factors including the nature of thematic analysis used and how the researcher characterizes a theme or codes, and our new information threshold remained relatively low.^26^

Among other limitations, only demographic questions related to the participants' involvement in the maternal health sector were included. Further data about participant characteristics, such as age, may be useful in future studies to provide a more detailed analysis about who engaged in the study. One interview participant was also an artist who contributed one piece of displayed work, which may have affected their objectivity.

Although the study was effective in engaging the general public, there was a relatively low attendance of the target audience groups ‘health policymakers’ and ‘third‐sector organization members’ despite being invited. This could possibly be due to logistics of location and time. It could therefore be advantageous in the future to upscale networking with key stakeholders and increase personal invitations to reach the full remit of the intended target population, particularly maternal and child health professionals. It may also be worthwhile linking ‘Birthing a Better Future’ together with large campaigns around health and social matters with a focus on 1001 days to broaden the audience. We also note that the highest yields of questionnaires were on the days that a researcher was present. Therefore, it may be useful to have a researcher or facilitator present on more days during the exhibition for further studies, or to use other forms of signposting, alongside other steps to avoid sampling bias. Enhancing the evaluation by providing opportunities for participants to feedback after they have left the event, and identifying more opportunities for in‐depth interviews either face‐to‐face or by phone following the event would increase our understanding on how to further improve such campaigns.

## 5 CONCLUSION

Overall, we found that a co‐created interdisciplinary multimedia exhibition is a valuable component of public health communication strategy. We identified a number of learning points, including engaging early with the target audience to shape the content and location of future exhibitions to their needs. For future studies, methods to engage a larger sample of individuals should be considered in addition to developing more rigorous methods to evaluate the effectiveness of such public health‐related complex interventions.

## DISCLOSURE

The authors report no conflict of interest.

## AUTHOR CONTRIBUTIONS

LM and ML conceived of the study. MAC and JO designed and piloted data collection tools. ML and LM reviewed the data collection tools. MAC, JO and VK led the primary data collection. MAC, JO, ML and ECA contributed substantially to the analysis. AF and AB contributed to the design of the work. ML, ECA, MAC and JO completed the initial manuscript draft. All other authors contributed to revising the draft and gave final approval of the version to be published and agreed to be accountable for the work.

## Data Availability

The data that support the findings of this study are available from the corresponding author upon reasonable request.

## APPENDIX 1

1. Sample of the pilot interview prompts

1. Sample of the final interview prompt

1. Sample of the pilot questionnaire

1. Sample of the final questionnaire
